# Oligomannose-Type Glycan Processing in the Endoplasmic Reticulum and Its Importance in Misfolding Diseases

**DOI:** 10.3390/biology11020199

**Published:** 2022-01-27

**Authors:** Taiki Kuribara, Kiichiro Totani

**Affiliations:** Department of Materials and Life Science, Seikei University, Musashino-shi, Tokyo 180-8633, Japan; tkuribara@st.seikei.ac.jp

**Keywords:** glycoprotein folding, misfolding diseases, glycan processing, reconstructed glycan profile

## Abstract

**Simple Summary:**

Glycans play many roles in biological processes. For instance, they mediate cell–cell interaction, viral infection, and protein folding of glycoproteins. Glycoprotein folding in the endoplasmic reticulum (ER) is closely related to the onset of diseases such as misfolding diseases caused by accumulation of misfolded proteins in the ER. In this review, we focused on oligomannose-type glycan processing in the ER, which has central roles in glycoprotein folding in the ER, and we summarise relationship between oligomannose-type glycan processing and misfolding diseases arising from the disruption of ER homeostasis.

**Abstract:**

Glycoprotein folding plays a critical role in sorting glycoprotein secretion and degradation in the endoplasmic reticulum (ER). Furthermore, relationships between glycoprotein folding and several diseases, such as type 2 diabetes and various neurodegenerative disorders, are indicated. Patients’ cells with type 2 diabetes, and various neurodegenerative disorders induce ER stress, against which the cells utilize the unfolded protein response for protection. However, in some cases, chronic and/or massive ER stress causes critical damage to cells, leading to the onset of ER stress-related diseases, which are categorized into misfolding diseases. Accumulation of misfolded proteins may be a cause of ER stress, in this respect, perturbation of oligomannose-type glycan processing in the ER may occur. A great number of studies indicate the relationships between ER stress and misfolding diseases, while little evidence has been reported on the connection between oligomannose-type glycan processing and misfolding diseases. In this review, we summarize alteration of oligomannose-type glycan processing in several ER stress-related diseases, especially misfolding diseases and show the possibility of these alteration of oligomannose-type glycan processing as indicators of diseases.

## 1. Introduction

Glycans play critical roles in mammals [1] and are categorized as *O*- and *N*-linked glycans. Specifically, they function as a major signal of biological processes, such as cell–cell interaction, viral infection, protein folding of glycoprotein [2]. Among these, the *N*-glycan-mediated glycoprotein folding mechanism in the endoplasmic reticulum (ER) is critical for proper glycoprotein folding and functioning in the desired cellular compartment. However, misfolded glycoproteins should be degraded to maintain cellular homeostasis, because the accumulation of misfolded glycoproteins in the ER causes stress to cells, namely ER stress. Therefore, cells have a quality control system named ER glycoprotein quality control (glycoprotein ERQC) (described in more detail in Section 2) [3,4,5,6].

This system enables the secretion of folded glycoprotein to the Golgi apparatus and the removal of misfolded glycoproteins from the ER to the cytosol for degradation. In this system, the glycan attached on proteins is an intriguing molecule in various aspects. First, *N*-glycans, especially oligomannose-type glycans, on protein scaffolds act as sorting signals for glycoprotein secretion and degradation. Thus, these oligomannose-type glycans may reflect information about protein scaffold-folding states. Second, the operating status of oligomannose-type glycan processing in the ER may reflect various cellular conditions, such as diseases caused by accumulation of (glyco)proteins in the ER. With respect to these, this review summarises glycoprotein ERQC and various diseases, and discusses the possibility for alteration of operating status of oligomannose-type glycan processing in misfolding diseases. 

## 2. Glycan Roles in Glycoprotein Biosynthesis and Glycoprotein ERQC

**Figure 1:**
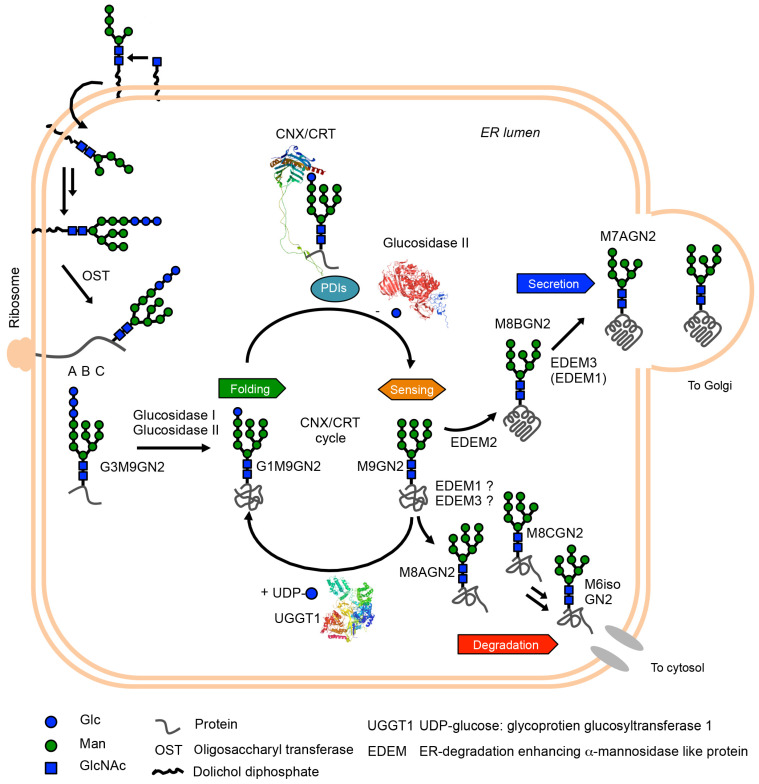
Schematic representation of glycoprotein ERQC. First, mature G3M9GN2 glycan is attached to newly ribosome-synthesized polypeptide by the action of oligosaccharyltransferase (OST) complex. Subsequently, glucosidase I and II sequentially trim two glucose residues, yielding G1M9GN2 glycoproteins. This glycan structure is critical to protein folding of glycoproteins. Namely, calnexin (CNX) and its soluble homologue calreticulin (CRT) capture G1M9GN2 polypeptides by its lectin activity, and their complex with protein disulfide isomerases (PDIs) accelerates protein folding of G1M9GN2 glycoproteins. Next, outermost glucose residues of G1M9GN2 glycoproteins are trimmed by glucosidase II, and folding states of resultant M9GN2 glycoproteins are checked by a folding sensor enzyme (UGGT1). Once UGGT1 determined folding intermediates, the enzyme re-transfers a single glucose residue to A-branch of M9GN2-glycoproteins. This regenerating G1M9GN2 folding intermediate obtains a refolding opportunity by the CNX/CRT-PDIs complex. This cycle system, referred to as the CNX/CRT cycle, can maximize protein folding in the ER. After the sensing process, M9GN2 glycoproteins become substrate of mannosidases in the ER (EDEM1, EDEM2 and EDEM3). A major pathway in mammalian cells is mannose-trimming of M8BGN2 from M9GN2 by the action of EDEM2, and subsequent mannose-trimmings by EDEM3/EDEM1 generate M7AGN2 and further trimmed oligomannose-type glycan. On the other hand, whether M8AGN2 and M8CGN2-glycoproteins are generated in the ER is still under investigation. Although it is believed that the M8AGN2 and M8CGN2 are mainly generated by the action of Golgi mannosidases in vivo, based on the in vitro branch specificity of EDEMs [18,19], EDEM1 or EDEM3 may mediate the process. However, further evidence in vivo and in vitro is necessary. At least, results of total cellular glycan analysis of MAN1B1-CDG patient indicate that M8AGN2 exists and M6isoGN2 is accumulating in patients’ cells, though where these glycans are produced originally is unclear [20]. Collectively, based on in vitro ER lectins and enzymes specificity [21,22,23], it is considered that M8BGN2 and M8AGN2 can act as secretion signal and degradation signal precursor, respectively. To consider this possibility in vivo, further evidence is necessary. 3D structures of CRT, glucosidase II and UGGT1 are visualized by Waals software. The PDB IDs of the structures are 5F0E for glucosidase II [24], 6ENY for CRT [25], and 5N2J for UGGT1 [26].

It is considered that approximately 50% of total ER proteins include oligomannose-type glycans in the asparagine (Asn) residue in the consensus sequence of glycosylation site (Asn-X-Ser/Thr X-any amino acid except proline) [7]. When focused on the glycan maturation step, the glycan is biosynthesized in the ER membrane’s outer and inner leaflets by the action of several glycosyltransferases [8]. After the glycan maturation process in healthy mammalian cells, glycan (Glc_3_Man_9_GlcNAc_2_: G3M9GN2 in eukaryotes, which are species-dependent structural variations [9]) is co-translationally or post-translationally transferred to the newly synthesized polypeptides by the action of oligosaccharyltransferase (OST) complexes containing STT3A or STT3B as the catalytic subunits and six common subunits (RPN1, RPN2, DAD1, OST48, OST4, and TMEM258) and specific subunits (DC2/OSTC and KCP2 for OST containing STT3A, and TUSC3 and MAGT1 for OST containing STT3B) [10,11,12]. The glycopolypeptides are simultaneously folded during the transglycosylation reaction by the OST complexes. Interestingly, in the case of certain diseases, such as congenital disorders of glycosylation (CDGs) [13,14], glycan maturation reaction is known to be insufficient due to mutation of genes encoding glycan biosynthesis and glycan processing proteins. This may cause the production of an aberrant glycan and reduce efficiency of glycoprotein folding in the ER [15]. From this perspective, the glycan on newly synthesized polypeptides is a critical signal for the protein folding of glycoproteins. In addition, 1-deoxymannojirycin [16], which is an inhibitor of mannosidases in the ER, can induce ER stress [17]. The study also showed that ER stress induces the apoptosis of hepatocarcinoma cell 7721 [17]. This suggests that the glycan processing plays a critical role in glycoprotein ERQC and maintaining ER homeostasis. A schematic view of glycoprotein ERQC is given in Figure 1.

After production of G3M9GN2-polypeptides in mammalian cells, the outermost glucose residue of G3M9GN2 is rapidly trimmed by the action of ER glucosidase I, which is also known as mannosyl-oligosaccharide glucosidase I [27]. Subsequently, further single glucose residue of G2M9GN2-glycopeptides are trimmed by the action of ER glucosidase II [24,28], producing G1M9GN2-glycopeptides. The resultant G1M9GN2-polypeptides are assisted in polypeptide folding by lectin chaperones, namely calnexin/calreticulin (CNX/CRT) [29,30] and protein disulphide isomerase family (ER-resident protein 57 [31], ER-resident protein 29 [32], cyclophilin B [33]) complex. The lectin chaperones capture G1M9GN2-polypeptides and aid glycoprotein folding. Additionally, a recent study suggested that the chaperone activity of CRT depends on hydrophobicity of aglycone of synthetic glycan substrates [34]. Furthermore, the complex formation accelerates the folding of G1M9GN2 polypeptide moiety by rapidly exchanging disulphide bond formation for efficient protein folding [35]. As G1M9GN2-bearing polypeptides are folded, CNX/CRT releases G1M9GN2-glycoproteins that are trimmed from the outermost glucose residue of G1M9GN2 by ER glucosidase II [36], generating M9GN2-glycoproteins. ER glucosidase II cannot access unreleased G1M9GN2 glycoproteins due to steric clashes between G1M9GN2-bound CNX/CRT and ER glucosidase II. Generated M9GN2-glycoproteins are recognized by the folding sensor enzyme uridine diphosphate-glucose: glycoprotein glucosyltransferase 1 (UGGT1) [37,38], and are monitored its folding states by UGGT1’s sensing ability [39]. When UGGT1 detects insufficient folding (folding intermediates), the enzyme re-transfers a single glucose residue to the A-branch of M9GN2-glycoproteins, regenerating G1M9GN2-glycoproteins. This allows refolding of G1M9GN2-folding intermediates. The CNX/CRT cycle, which refers to the glucose residue-dependent folding acceleration, check, and refolding system, is a fundamental mechanism in glycoprotein ERQC [40]. 

The folded or misfolded M9GN2-glycoproteins are considered to the substrate for α-1,2 mannosidases in the ER [ER-degradation enhancing α-1,2 mannosidase-like protein (EDEM1, EDEM2, and EDEM3)] and UGGT1. M9GN2-folded glycoproteins, a poor substrate of UGGT1, are trimmed by EDEM family proteins. Moreover, misfolded M9GN2-glycoproteins are known as poor-substrate of UGGT1. When misfolded M9GN2-glycoproteins are generated in the ER, EDEM family proteins can trim misfolded M9GN2-glycoproteins and accelerate its degradation. Reportedly, only M9GN2-folding intermediates become a good substrate for UGGT1 rather than folded or misfolded M9GN2-glycoprotein substrates [38,39]. Considering these, competition between the UGGT1, and EDEM proteins exist for folded, folding intermediates, and misfolded M9GN2-glycoproteins; further experimental evidence is needed. Taken together, these characteristics toward M9GN2-glycoproteins may at least explain the factor releasing the folded and/or misfolded M9GN2-glycoproteins from UGGT1, and EDEM family proteins trim mannose residues from folded and/or misfolded M9GN2-glycoproteins. The mannose-trimmings of M9GN2-glycoproteins contribute to sorting glycoprotein secretion and degradation. The interpretation of EDEM family proteins significantly changed in the last 20 years. In the 1990s, α-1,2 mannosidase in the ER (ERManI, also known as MAN1B1) was discovered [41,42] and has been reported as responsible for the production of M8BGN2 from M9GN2-substrates in vitro. In 2001, another α-1,2 mannosidase-like protein (EDEM) was discovered [43], while α-1,2 mannosidase activity was not detected; thus, EDEM was believed to be an M8BGN2 recognizing lectin at the time [44]. Later studies discovered EDEM homologues (EDEM2 [45] and EDEM3 [46]), and in vivo weak α-1,2 mannosidase activity of EDEM1 and EDEM3 was revealed [46,47]. Furthermore, in vitro weak EDEM1 and EDEM2 activity towards free N-glycan and glycoproteins was revealed [48]. Recent studies have demonstrated that all EDEMs possess sufficient α-1,2 mannosidase activity in vitro [18], and an especially significant finding is the evidence of high EDEM2’s α-1,2 mannosidase activity by stable disulphide bond formation with TXNDC11 in vitro [49]. 

Conversely, MAN1B1 is still a controversial α-1,2 mannosidase in terms of cell localization. Firstly, the localization of overexpressed MAN1B1 was reported as ER. Later studies showed endogenous MAN1B1 localized, such as the Golgi apparatus [18,50,51] and quality control vesicle [52]. Collectively, major α-1,2 mannosidases in the ER are now believed to be EDEMs, while MAN1B1 may play certain roles in the Golgi apparatus rather than ER. Although the interpretation of EDEMs significantly improved, the entire mannose-trimming pathway, including minor pathway, remains unclear. When we focused on their acting point on M9GN2, four α-1,2 mannoside linkages exist. Although EDEM2 dominantly trims outermost mannose residue of B-branch from M9GN2 then EDEM3/EDEM1 (EDEM3 is dominant than EDEM1) trim residual A or C-branch mannose residues from M8BGN2 [6,18,49], perhaps the trimming ability towards A or C-branch by EDEM3/EDEM1 may adopt M9GN2 glycans. Indeed, oligomannose-type glycan analyses of total cell lysate showed small-scale but important production of M8AGN2 and M8CGN2 [53]. These glycans would be produced by Golgi α-1,2 mannosidase family proteins [54,55], while another possibility is that ER mannosidases may produce M8AGN2 or M8CGN2 glycans. Further studies on this possibility in cellular levels are necessary. About this possibility in vitro, we previously reported the analysis of mannose trimming with synthetic glycan substrates and ER fractions derived from animal tissue in the presence of various glycosidase inhibitors to investigate selective inhibitors of α-1,2 mannosidase in the ER. We identified two reciprocally selective inhibitors and applied them to mannose-trimming pathway analysis in vitro. That study revealed that two independent mannose-trimming pathways mediated by the production of M8BGN2 or M8AGN2 from M9GN2 exist using selective inhibitors of α-1,2 mannosidase in the ER [19]. Considering the lectin and enzyme specificity of secretion and degradation-related proteins [21,22,23], M8BGN2 and M8AGN2 function as a secretion signal and degradation signal precursor, respectively. Indeed, the accumulation of M6isoGN2, which is a twice-trimmed product from M8AGN2, was observed in MAN1B1-CDG patients, indicating that M8AGN2 and further trimmed oligomannose-type glycans may be toxic to cells [20]. Although further evidence is required, these results indicate that M8BGN2 and M8AGN2 can act as secretion and degradation signals of glycoproteins, respectively. 

Taken together, glycoprotein ERQC is important for sorting secretion or degradation glycoproteins and some of specific glycan isomers could act as signals of folding, secretion and degradation of glycoproteins.

## 3. Oligomannose-Type Glycan Processing in Glycoprotein ERQC and Diseases

Even though cells have glycan processing (glucose trimmings, glucose transfer and mannose trimmings) system for sorting folded or misfolded glycoproteins, approximately 30% of newly synthesized (glyco)proteins are intrinsically misfolded and degraded by proteasomes [56]. Therefore, degradation of misfolded glycoproteins is one of the key factors maintaining ER homeostasis. Nevertheless, it has been reported that about 15% of proteins that are synthesized from genetic code will have at least one misincorporated amino acid [57]. In addition, misincorporation disrupts the correct folding of erroneous polypeptides. Furthermore, certain proteins tend to undergo spontaneous unfolding; collectively, protein folding is an error-prone process despite existence of glycoprotein ERQC. Thus, accumulation of misfolded (glyco)proteins is often observed, and the misfolded (glyco)proteins may induce ER stress. Massive ER stress causes apoptosis and is closely related to several diseases (detailed in Section 3.1). To cope with the stress, cells have a protective mechanism against ER stress named unfolded protein response (UPR) for clearance of accumulated misfolded (glyco)proteins from ER. In UPR, expression of several glycoprotein ERQC components are altered. This may suggest alteration of operating status of oligomannose-type glycan processing is occurred in ER stress condition. However, the connection between the glycan processing and ER stress related diseases are unclear. Therefore, we demonstrate the perturbation of oligomannose-type glycan processing in glycoprotein ERQC in some kind of misfolding diseases (detailed in Section 3.2).

### 3.1. ER Stress and Misfolding Diseases

Accumulation of misfolded (glyco)proteins causes ER stress. Therefore, cells have a protective mechanism, called UPR, for accumulated misfolded non-glycoproteins and glycoproteins. UPR is a major mechanism for maintaining ER homeostasis and has already identified three main pathways [58]. In brief, translation of mRNA is suppressed by activating the protein kinase RNA-like ER kinase (PERK) pathway. Activating PERK leads to the phosphorylation of eukaryotic translation initiation factor-2α, which inhibits binding of preinitiation complex to the cap structure of mRNAs, resulting in the suppression of protein translation. Furthermore, the up-regulation of certain genes such as activating transcription factor 4 induces an antioxidant response and promotes protein folding capacity. The stimulation of the activating transcription factor 6 (ATF6) pathway and the up-regulation of ER chaperones induce increased folding capacity and accelerate protein folding in the ER. The inositol-requiring protein 1 α (IRE1) pathway, which is responsible for up-regulating protein folding-related proteins, ER-associated degradation (ERAD)-related proteins, and lipid biosynthesis-related proteins, is activated after the ATF6 pathway to refold and/or remove misfolded glycoproteins [59]. However, chronic or massive ER stress causes severe conditions that make the cells no longer survive. Therefore, these cells are cleared from tissues by activating apoptosis signals. The UPR is widely activated in several diseases, such as type 2 diabetes and a series of neurodegenerative disorders [Alzheimer’s disease (AD), Parkinson’s disease (PD), Huntington’s disease (HD), amyotrophic lateral sclerosis (ALS) and prion diseases]. Several classifications of ER stress-related diseases, including above-mentioned diseases, have reported [60]. For instance, genetic disorders and neurodegenerative disorders are mainly caused by loss of function or misfolding of mutant proteins. Environmental or lifestyle insults such as an excess of nutrients or inflammation induce ER stress, and these are causative to neurodegenerative disorders and metabolic and inflammatory diseases. In particular, accumulation of etiological proteins by genetic mutation or misfolding is categorized into misfolding diseases. The common features are misfolding of (glyco)proteins and forming toxic aggregates that induce ER stress in the original tissue, while disease-associated proteins are quite different. Furthermore, activating UPR pathways vary depending on the disease. In this section, among various misfolding diseases reported, we focused on age-related diseases because the world is shifting to an ageing society, and improvement of patients’ quality of life is an urgent need. The most familiar age-related diseases are type 2 diabetes and neurodegenerative disorders, and we discuss these misfolding diseases. 

#### 3.1.1. Type 2 Diabetes

Obesity exacerbates obese type 2 diabetes. Indeed, in high-fat diet-induced genetic (*ob*/*ob*) mouse models, obesity causes ER stress and it is considered that obesity contributes to the development of obese type 2 diabetes [61]. This study also revealed that ER stress is predominantly induced in liver and adipose tissues and inhibits insulin action in liver cells. This indicates that ER stress leads to the development of insulin resistance and is one of the progression factors to obese type 2 diabetes.

In addition to liver tissue, type 2 diabetes is well characterised in pancreatic β-cells. Major features are dysfunction of β-cells and loss of β-cells mass [62]. These may be explained by various stresses, including ER stress in the β-cells. Islet amyloid polypeptides (IAPP) in β-cells may induce cell toxicity, resulting in ER stress mediating one of the factors for β-cell apoptosis [63]. This may result in impaired insulin release and hyperglycaemia, developing type 2 diabetes. Huang et al., reported that IAPP induces CHOP, which is one of the apoptosis mediators in the PERK pathway [64]. Although CHOP is not the only mediator for apoptosis of the β-cells, these results indicate the relationships between IAPP and ER stress and that IAPP may be one of the associated proteins of type 2 diabetes. Proinsulin is another possibility of associated proteins in type 2 diabetes. Indeed, proinsulin misfolding is observed in an early event in the progression to type 2 diabetes [65]. Interestingly, it is characterised by olanzapine, a second-generation antipsychotic, which causes adverse side effects, including diabetes. A recent study demonstrated that olanzapine-induced diabetes is caused by olanzapine-induced proinsulin misfolding in β-cells [66]. Furthermore, these studies also suggest that the PERK pathway is activated in β-cells. Altogether, IAPP and proinsulin may contribute to type 2 diabetes by activating the PERK pathway, and these studies highlight protein misfolding involved in type 2 diabetes.

#### 3.1.2. Neurodegenerative Disorders

In neurodegenerative disorders, such as AD, PD, HD, ALS and prion protein disease, several misfolded proteins are observed, such as neurotoxic oligomers of the amyloid β-peptides (Aβ) and Tau in AD, α-synuclein in PD, a polyglutamine (polyQ) extended huntingtin in HD, superoxide dismutase (SOD1), Tar-DNA binding protein (TDP-43) and fused in sarcoma (FUS) in ALS, and prion protein (PrP) in prion diseases. Although the roles of these proteins in neurodegeneration are diverse, ER stress is induced in all diseases. In this section, we partially introduce the relationships between disease-associated proteins and ER stress from excellent reviews, in which more details of the pathological mechanism of each neurodegenerative disorder have been described [67,68,69,70,71].

##### Alzheimer’s Diseases (AD)

In AD [67], it is characterized as an indirect Aβ contribution for ER stress, because interaction with cytosolic oligomers of Aβ and neuronal N-methyl-D-aspartate receptors can disrupt cytosolic calcium balance and after cell signalling, leading to ER stress-dependent cell death [72]. Furthermore, in the case of rare familial AD forms, Aβ accumulation in ER was observed, indicating that Aβ may directly contribute to ER stress induction [73]. In contrast to Aβ, soluble Tau in cytosol inhibit ERAD activity, triggering accumulation of misfolded proteins and causing ER stress [74]. Therefore, in AD, Aβ and Tau contribute to evoking UPR pathways. Although all UPR pathway activations have been known, PERK and IRE1 pathways are well characterized. For instance, the PERK pathway first induces protein translation inhibition and the activation increases the expression of β-amyloid precursor protein cleaving enzyme 1 (BACE1), leading Aβ production [75]. Sustained PERK signalling may cause neuronal loss through apoptosis signals [67], thus the PERK pathway may have a time-dependent response for AD progression. Furthermore, spliced X-box binding protein 1 (XBP1), a major signal transducer in the IRE1 pathway, increases the degradation rate of key AD proteins such as BACE1 and phosphorylated tau. However, sustained IRE1 signalling might induce pro-apoptotic activity by regulated IRE1-dependent decay rather than XBP1s expression. Interestingly, Gerakis et al., proposed connections between UPR and Alzheimer’s disease lesions; they indicated that protein aggregation and accumulation is the first step in Alzheimer’s disease [67]. Thus, the operating status of glycoprotein ERQC may also alter by changing the expression level of ERQC components in an early stage of Alzheimer’s disease.

##### Parkinson’s Disease (PD)

In PD [68], Overexpression of human α-synuclein and its A53T mutant exhibit toxicity and cause ER stress [76,77]. Although α-synuclein is not an ER-resident protein, α-synuclein-mediated ER stress may be caused either directly or indirectly through several cell signalling pathways [68]. To summarize, toxic α-synuclein can bind synaptic vesicles and biological membranes, including ER, thus affecting intracellular protein trafficking [78,79,80]. Then, toxic α-synuclein and/or its aggregates may induce ER stress. Among all UPR pathways, the PERK pathway may contribute to neurodegeneration. For instance, an overexpressed A53T mutant of α-synuclein in PC12 cells causes the phosphorylation of eukaryotic translation initiation factor-2α and induces CHOP expression, which indicates that the PERK pathway was activated [76]. Furthermore, inhibition of XBP1 protein expression in mice induced chronic ER stress and neurodegeneration, while recovery of XBP1 level by gene therapy protected neuronal cells [81]. Thus, the IRE1 pathway plays a critical role in neuronal survival. Furthermore, ATF6 knockdown in mice exacerbated neurotoxicity after treatment with dopaminergic neurotoxin [82]. These results speculate about the expression alteration of ERQC components; however, scant evidence is reported.

##### Huntington’s Disease (HD)

In HD [69], patients have a mutation of huntingtin then HD is a genetic disorder. The mutant has polyQ [83] and is prone to misfolding and aggregation in the cytosol. This mutant huntingtin or the aggregate accumulate, leading to ERAD inhibition [84,85]. Interestingly, several studies showed that oligomers of mutant huntingtin are more cytotoxic than their aggregate forms [85,86]. Furthermore, ERAD substrate accumulation was observed and all three pathways of UPR were activated. In addition, activation of the IRE1 pathway was reported in vivo [87]. These results suggest that ERAD inhibition by toxic huntingtin oligomers induces ER stress and the activation of UPR. Furthermore, in the ER stress condition, increased expression of protein disulfide isomerases (PDIs), an important protein for protein folding in the ER, was reported [69,84]. This may indicate at least that the activity of PDIs involved in (glyco)protein folding in the ER is altered in HD.

##### Amyotrophic Lateral Sclerosis (ALS)

In ALS [70], several proteins have characterized ALS-associated proteins, such as SOD1, TDP-43 and FUS. The mutants of these three proteins are prone to misfolding and aggregation, inducing ER stress. For instance, mutant SOD1 interacts with Derlin-1, which is important for ERAD, inducing ER stress [88]. Similar to SOD1, mutant TDP-43 and FUS activate UPR from the cytosol. CHOP induction was observed and this implicates the activation of the PERK pathway, leading to apoptosis in neuronal cells expressing mutant SOD1 [89]. Interestingly, XBP-1 deficiency can delay ALS disease onset in mutant SOD1 mice by increasing autophagy [90]. This indicates IRE1 pathway can provide a protective effect for ALS. In the case of TDP-43, overexpression of wild-type and mutant TDP-43 induced ER stress by activating XBP-1 and ATF6 pathways [91]. Furthermore, cytosolic FUS induced ER stress and colocalized with PDI [92]. Interestingly, reducing CRT levels in G93A mutant SOD1 mice triggered muscle denervation and motor neuron degeneration in mice [93,94]. These results may indicate that the altered expression of glycoprotein ERQC component in ALS is plausible.

##### Prion Diseases

In prion diseases [71], PrP is prone to misfolding by abnormal folding because the α-helix rich form of PrP that is frequently observed in normal cellular conditions converts into misfolding prone β-sheet conformation [71]. The conformational changes alter its properties such as solubility and resistance to proteases. Thus, the accumulation of abnormal conformation of PrPs might be associated with ER stress. Hetz et al., reported that highly purified abnormal conformations of PrPs induce ER stress and apoptosis in vitro [95]. The PERK pathway was activated in the hippocampi of PrP-infected and overexpressing mice [96]. In the mouse model, PrP accumulation activated the PERK pathway and resulted in neurodegeneration [96]. Furthermore, PERK pathway inhibition by several pharmacological inhibitors provides neuroprotective activity [97,98]. These studies indicate the importance of the PERK pathway in prion diseases. Additionally, the dominant negative form of IRE1 or XBP1 significantly increased PrP aggregation, while the active mutant form of XBP1 and ATF6 had the opposite effect [99]. These results suggest that all UPR pathways are activated in prion diseases. Furthermore, Yedidia et al., reported approximately 10% of normal conformation of PrP is intrinsically misfolded and degraded by the ERAD pathway [100]. This may imply that PrP itself is involved in the perturbation of glycoprotein ERQC.

Collectively, relationships between ER stress and ER stress-related diseases—especially misfolding diseases—have been demonstrated in a large body of studies. Among them, the involvement of UPR pathways in type 2 diabetes and neurodegenerative diseases is partially demonstrated. In these diseases, toxic proteins lead to misfolding of proteins in the ER, inducing ER stress. Accumulating misfolded proteins activates all three UPR pathways, and may lead to changing expression levels of glycoprotein ERQC components. As described above, although some circumstantial evidence for this possibility has been reported [76,81,84,92,93,94,100], no direct connections between misfolding diseases and glycoprotein ERQC have been reported. Considering these, altered oligomannose-type glycan processing in glycoprotein ERQC might occur; however, this is not well understood.

### 3.2. Oligomannose-Type Glycan Processing and Misfolding Disease

**Figure 2:**
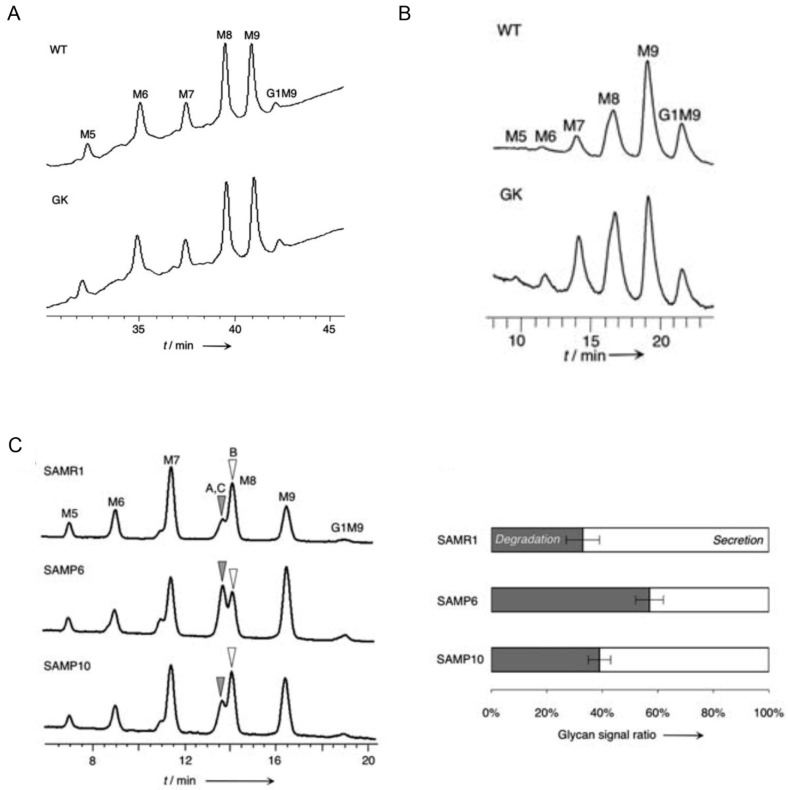
Importance of reconstructed glycan profiles in terms of detecting perturbation of glycoprotein ERQC. (**A**) Total glycan-processing analysis from isolated ER lysate did not detect differences in healthy (WT) and non-obese type 2 diabetes rat models (GK). Direct glycan profile reflects in situ glycan processing states in isolated ER lysate. In line with previous findings, contamination of ER-Golgi recycling glycoproteins was detected. This is one of reasons why perturbation of glycan profile is masked by the direct glycan profile method. (**B**) Compared to direct glycan profile, reconstructed glycan profiles can obtain time-dependent glycan profiles, because ER glycan processing is reconstructed in test tube, as described in Section 3.2. The method enables detection of slight differences of activity of glycan processing enzymes. This enables detection of the perturbation of glycoprotein ERQC. (**C**) Reconstructed glycan profiles in mouse models of misfolding diseases. The important findings are M8AGN2 and M8CGN2, degradation signals, increased in these mouse models. These figures are adapted from ref [101].

In this section, we demonstrate whether the alteration in glycan processing in the ER can reflect disease states, such as type 2 diabetes and neurodegenerative disorders. In this respect, a comparison of precise glycan-processing states from healthy and disease samples is needed. Overall, a comparison of glycan-processing analysis is conducted in serum or total cellular proteins. However, at least oligomannose-type glycans mainly exist in ER. Furthermore, they are common intermediates in secretory glycoprotein’s glycan. Therefore, perturbation of oligomannose-type glycans may occur in patients’ ER at the early stage of diseases. However, oligomannose-type glycans convert to several types of other N-glycans in Golgi apparatus (complex- and hybrid-type N-glycans), alteration of oligomannose-type glycans in patients’ samples is often overlooked. To precisely analyse the glycans in ER, total glycan-processing analysis (glycan profiles) using isolated ER is most suitable because isolated ER can reflect the exact glycan profiles in the ER. For this reason, we previously tried acquiring glycan profiles in isolated ER from animal model tissues of misfolding disease model. However, the total glycan profiles from isolated ER were not different from healthy controls (Figure 2A) [101]. This suggests that the direct glycan profile cannot reflect the nature of ER glycan profile, because the recycling of glycoproteins between ER- and Golgi apparatus may mask differences of perturbation of glycan profiles in disease sample.

To overcome this problem, we established a novel glycan-processing analysis system named the reconstructed glycan profile method [101]. The method has three main features: (1) ER fraction is derived from model animals as enzymatic reaction sources, (2) synthetic fluorescent-labelled glycan substrates is used to substrates [102], and (3) high-performance liquid chromatography for facilitating separation of various oligomannose-type glycans. With the ER fraction and the synthetic glycan substrates, we analysed in vitro operating status of glycan processing at various reaction times. If the alteration of the glycan profiles from healthy to disease-affected is observed, the differences will indicate the feature of diseases. Indeed, the glycan profiles are different in several misfolding diseases models. For instance, the non-obese type 2 diabetes model rat, Goto-Kakizaki rat, clearly showed the differences in glycan profiles (Figure 2B). Furthermore, reconstructed glycan profiles of osteoporosis and dementia (one of the neurodegenerative disorders) model mouse, senescence-accelerated mouse p6 (SAMP6) and SAMP10 showed increasing M8AGN2 and M8CGN2 production, respectively (Figure 2C). This suggests that mannose-trimming activity is somehow affected by disease conditions; especially, the accumulation of degradation signals of glycoproteins is interesting findings. Moreover, the study investigated the protein expression of the ER obtained from these disease model animals and the expression of some glycan processing-related proteins altered. Although the relationship between disease manifestation and the alteration of glycan processing in the ER is unclear at this stage, perturbation of glycoprotein ERQC obviously occurs in these diseases.

Recently, we have reported perturbation of CNX/CRT cycle in obesity and obese type 2 diabetes model rats, namely, Zucker fatty rat (ZF) and Zucker diabetic fatty rat (ZDF) [103,104] using the reconstruction approach. To summarize simply, all CNX/CRT cycle-related proteins’ expression and activity were reduced in ZF. All the protein’s expression increased in ZDF compared with ZF, while the enzymatic activity, specifically, the activity of UGGT1, reduced despite increasing UGGT1 expression in mRNA and protein levels. This indicates that UGGT1 may be misfolded in ZDF rats, although further study for this possibility is needed.

These findings suggest reconstructed glycan profiles as a novel method for reflecting the operating status of glycoprotein ERQC in some misfolding diseases. Further, we believe the method can apply to other models of misfolding diseases.

## 4. Conclusions

In this review, we summarize glycoprotein ERQC (referred to in Section 2) and the involvement of ER stress-related diseases, especially misfolding diseases (detailed in Section 3.1). Glycans attached on proteins in the ER play critical roles in folding, secretion, and degradation signals of glycoproteins; thus, glycan processing in the ER might reflect the ER status in various cellular conditions. To this hypothesis, we partially demonstrated the reconstructed glycan profile method as a possibility for detecting perturbation of glycoprotein ERQC in models of several misfolding diseases (detailed in Section 3.2).

Many issues remain to be addressed, such as how connections between perturbation of oligomannose-type glycan processing in the ER and the manifestation of several diseases exist, and the selection of original tissue based on misfolding diseases used in the reconstructed glycan profile. Nevertheless, the reconstructed glycan profile method is a novel strategy detecting masked slight perturbation of oligomannose-type glycan processing in glycoprotein ERQC and may demonstrate applicability for diagnostics of misfolding diseases. Collectively, these findings highlight the importance of oligomannose-type glycan processing in the ER not only for glycoprotein sorting but also as indicators of misfolding diseases.

## Data Availability

Not applicable.
